# Evaluation of non-response bias in a cohort study of World Trade Center terrorist attack survivors

**DOI:** 10.1186/s13104-015-0994-2

**Published:** 2015-02-15

**Authors:** Shengchao Yu, Robert M Brackbill, Steven D Stellman, Sharon Ghuman, Mark R Farfel

**Affiliations:** New York City Department of Health and Mental Hygiene, Long Island City, NY USA; Department of Epidemiology, Mailman School of Public Health, Columbia University, New York, NY USA

**Keywords:** Non-response, Bias, Disaster, 9/11 exposure, Probable posttraumatic stress disorder, Lower respiratory symptoms, Drop-ins, Drop-outs

## Abstract

**Background:**

Few longitudinal studies of disaster cohorts have assessed both non-response bias in prevalence estimates of health outcomes and in the estimates of associations between health outcomes and disaster exposures. We examined the factors associated with non-response and the possible non-response bias in prevalence estimates and association estimates in a longitudinal study of World Trade Center (WTC) terrorist attack survivors.

**Methods:**

In 2003–04, 71,434 enrollees completed the WTC Health Registry wave 1 health survey. This study is limited to 67,670 adults who were eligible for both wave 2 and wave 3 surveys in 2006–07 and 2011–12. We first compared the characteristics between wave 3 participants (wave 3 drop-ins and three-wave participants) and non-participants (wave 3 drop-outs and wave 1 only participants). We then examined potential non-response bias in prevalence estimates and in exposure-outcome association estimates by comparing one-time non-participants (wave 3 drop-ins and drop-outs) at the two follow-up surveys with three-wave participants.

**Results:**

Compared to wave 3 participants, non-participants were younger, more likely to be male, non-White, non-self enrolled, non-rescue or recovery worker, have lower household income, and less than post-graduate education. Enrollees’ wave 1 health status had little association with their wave 3 participation. None of the disaster exposure measures measured at wave 1 was associated with wave 3 non-participation. Wave 3 drop-outs and drop-ins (those who participated in only one of the two follow-up surveys) reported somewhat poorer health outcomes than the three-wave participants. For example, compared to three-wave participants, wave 3 drop-outs had a 1.4 times higher odds of reporting poor or fair health at wave 2 (95% CI 1.3-1.4). However, the associations between disaster exposures and health outcomes were not different significantly among wave 3 drop-outs/drop-ins as compared to three-wave participants.

**Conclusion:**

Our results show that, despite a downward bias in prevalence estimates of health outcomes, attrition from the WTC Health Registry follow-up studies does not lead to serious bias in associations between 9/11 disaster exposures and key health outcomes. These findings provide insight into the impact of non-response on associations between disaster exposures and health outcomes reported in longitudinal studies.

## Background

The assessment of long-term health outcomes among individuals exposed to the September 11, 2001, terrorist attack on the World Trade Center (WTC) in New York City poses epidemiological challenges that may more generally be encountered following large scale natural or man-made disasters. One such challenge is to identify and quantify long-term health outcomes in a cohort consisting of a heterogeneous mixture of distinct survivor groups. A potential consequence of this heterogeneity is differential response to follow-up data acquisition efforts that could bias estimates of disease incidence and prevalence as well as estimates of exposure-outcome associations.

The WTC Health Registry, a cohort study of 71,434 survivors of the September 11 attacks, is such a case, being a combination of first responders, lower Manhattan residents and area workers, tourists and other passersby, and children. Data were gathered in three separate surveys between 2003 and 2012. Among wave 1 respondents, response rates to the two adult follow-ups were 68% and 63% respectively. The WTC Health Registry is designed to be maintained for at least 20 years after 9/11/2001. To better understand the long-term health effects of the 9/11 disaster, and to better assess the post-disaster health care needs of survivors, a timely evaluation of selective participation in the follow-up studies and non-response bias in this unique disaster cohort is needed.

Non-response is a concern for epidemiological studies because the decision to respond to a study is rarely completely random, and in most cases it may be associated with characteristics of the study population, including demographics, socioeconomic status, health behaviors, and health conditions [1-9]. Any of these characteristics may also correlate with risk factors for the outcome under investigation; therefore the estimates of health outcomes and the estimated associations between exposure and outcome based solely on participants can be biased [10-12]. Potential bias resulting from decreasing participation rates, particularly in epidemiological cohort studies, makes it difficult to obtain accurate insights on the course of health problems and the health needs of a population at different time periods [13].

Compared to numerous studies that have examined factors associated with selective participation [3-9,14,15], relatively few studies have directly assessed non-response bias in terms of prevalence estimates of health outcomes or exposure-outcome associations. Evaluations of non-response bias in prevalence estimates of health outcomes have typically used as benchmarks data from health registries [1,16-20], other health surveys, or censuses [5], and then compared participants with non-participants. These studies usually found that participants were somewhat healthier than non-participants [1,4,12,17-19], but non-response bias in association or risk estimates of outcome variables by background characteristics or exposure variables were generally small or insignificant [1,4,11,16,17,20].

Although a well-recognized problem, whether non-response causes serious bias in disaster cohorts has rarely been examined. Two notable non-response bias studies concerned the Netherlands fireworks disaster [13,21]. Using multiple imputation to produce estimated plausible values for missing data due to non-response, these two studies indicated selective participation but did not find serious bias in prevalence estimates in health problems in either baseline or follow-up surveys [13,21]. Despite the advanced methodology and insightful findings from the Netherlands fireworks studies, more research on bias due to attrition among disaster cohorts is needed. Particularly, research that can utilize data from non-respondents without making a strong assumption about the randomness of the missing data, as in the Netherlands studies, is desired. To our knowledge, none of the previous disaster cohort studies has evaluated the possible non-response bias in estimates of associations between exposures and health outcomes. Reliable association estimates are particularly important in disaster cohort studies because they are essential for evaluating the public health burden of disasters accurately, and by extension, for ensuring that policies to meet the health needs of the survivors are developed based on reliable evidence. Sound knowledge of exposure-outcome associations is also beneficial for emergency preparedness planning.

The WTC Health Registry has two primary goals: to provide post-disaster prevalence estimates of health outcomes, and to estimate associations between disaster exposures and health outcomes. Both estimates are subject to non-response bias. Using substantial data collected from the three WTC Health Registry surveys completed thus far, in this study we assess non-response bias in prevalence estimates of health outcomes among enrollees in the Registry follow-up surveys, and the extent to which non-response may have affected estimates of associations between those outcomes and 9/11 exposures. In particular, we evaluate the possible impact of non-response bias on specific health outcomes that have been found to be elevated in the Registry population. Previous WTC Health Registry studies have found increased levels of health conditions, such as asthma [22,23], lower respiratory symptoms [24,25], posttraumatic stress symptoms [23,26], and co-occurring lower respiratory symptoms and probable posttraumatic stress disorder (PTSD) [27,28]. The present report focuses on respiratory symptoms and probable PTSD as indices of physical and mental health, given their high prevalence and the strong associations of both measures with 9/11 exposures documented in previous Registry studies [22-28]. We furthermore include self-assessed general health, which has been shown to be a strong predictor of subsequent mortality [29].

## Methods

### Background

The WTC Health Registry was established in 2002 to monitor the long-term physical and mental health of people exposed to the September 11, 2001 terrorist attack on the World Trade Center in New York City. It is the largest post-disaster registry in US history that prospectively follows a diverse population of exposed individuals. In 2003–04, the WTC Health Registry conducted a health survey with 71,434 enrollees. In 2006–07 and 2011–12, the Registry conducted two waves of follow-up health surveys among eligible wave 1 enrollees. All participants gave verbal informed consent to participate in the Registry. The US Centers for Disease Control and Prevention and the New York City Department of Health and Mental Hygiene institutional review boards approved the Registry protocol, including use of the data.

### Study population

**Figure 1:**
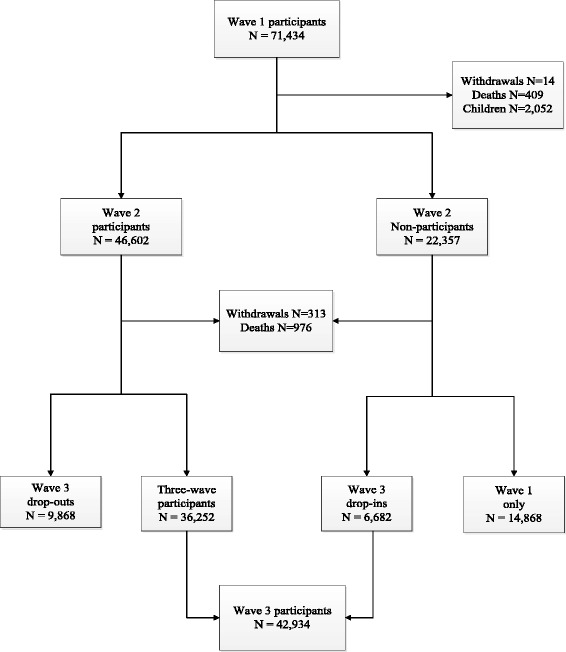
**Flow chart of World Trade Center Health Registry adult study population.**

A detailed description of WTC Health Registry has been reported previously [23,30]. In brief, at the enrollment stage, individuals were identified through lists obtained from lower Manhattan area employers and government agencies (“list-identified”) or using media campaigns that encouraged WTC-exposed individuals to contact the Registry for eligibility screening (“self-identified”). In 2003–04 a total of 71,434 eligible individuals, also called Registry enrollees, completed the wave 1 interview by phone (95%) or in person (5%). With the exception of decedents and those who withdrew from the Registry since wave 1, the entire adult cohort of the Registry was invited to participate in the wave 2 and wave 3 adult surveys in 2006–07 and in 2011–12. In total, 68,959 enrollees and 67,670 enrollees were eligible for inclusion in wave 2 and 3 respectively. This analysis is limited to the 67,670 enrollees eligible for both wave 2 and 3 adult surveys. Figure 1 shows the number of enrollees eligible for wave 3 in relation to their participation in earlier surveys: 36,252 individuals completed all three waves of the Registry surveys (“three-wave participants”); 9,868 are in the “wave 3 drop-out” group (enrollees who completed waves 1 and 2 but not wave 3); 6,682 are in the “wave 3 drop-in” group (enrollees who completed wave 1, did not participate in wave 2, but completed wave 3); and 14,868 individuals participated in the wave 1 survey only.

### Follow-up survey methods

Data were obtained from enrollees at both wave 2 and wave 3 using targeted outreach and three data collection modes in the following temporal order: web, a mailed paper survey, and telephone. Wave 2 data collection methods were described elsewhere [23]. For wave 3, starting in June 2011, 36,356 enrollees with valid e-mail addresses received an electronic invitation to access the web survey. After 9 subsequent email reminders, all non-responders to the e-mail invitation were mailed paper questionnaires; the web survey remained available for them. Four additional email reminders, 2 subsequent rounds of mailed paper surveys, and 3 postcard reminders were sent to enrollees who had not responded to the initial web survey invitation. Paper questionnaires were mailed to enrollees who did not have an email address on file beginning in July 2011. Two additional rounds of paper questionnaires and 3 postcard reminders were mailed to non-respondents in this group.

Beginning in September 2011, a six-month effort was made to contact web and paper non-respondents by telephone and to administer the survey by Computer Assisted Telephone Interviewing (CATI) to those successfully reached. Because of the much higher cost of CATI data acquisition relative to web or mail, CATI was directed at selected subgroups of enrollees. During the first five months we focused on enrollees who had participated in both wave 1 and wave 2, on the assumption that these individuals would be more likely to participate in wave 3 than wave 2 drop-outs. This strategy also addressed the desirability of having health assessments at three points in time for analytical studies of the time course of outcomes such as PTSD. During the final month, CATI efforts were expanded to include wave 2 drop-outs. It should be emphasized that throughout the 10-month data gathering period efforts were continuously made to provide every enrollee with an opportunity to respond by web or mail. A total of 6,381 surveys, or 15% of the total, were completed by phone, 19,170 (45%) by web and 17,383 (40%) by mail (total N = 42,934, response rate = 63%).

### Study variables

#### Population characteristics

The wave 1 survey provided data on demographic characteristics (sex, age, race, household income in 2002, and education), eligibility group, and recruitment source (list-identified vs. self-identified). Eligibility group is a mutually exclusive and hierarchical variable with rescue and recovery workers first, followed by lower Manhattan residents, and lower Manhattan area workers and passersby on September 11 [30].

#### Disaster exposures

Disaster exposure data were obtained in the wave 1 survey. Three measures of exposure are used: witnessing three or more traumatic or horrific events (seeing an airplane hitting the WTC towers, a building collapsing, people running away from a cloud of smoke, anyone injured or killed, or people falling or jumping from WTC towers), sustaining one or more of five listed injury types (cut, sprain or strain, burn, fracture or dislocation, or head injury) due to the WTC attack, and being caught in the dust or debris cloud on 9/11. These three measures were asked of all Registry enrollees.

#### Physical and mental health status

Enrollees reported their pre- and post-disaster health at wave 1. We chose three wave 1 health indicators to examine whether the baseline health status was associated with follow-up survey participation: 1) new or worsening respiratory symptoms since 9/11, defined as having developed or having worsened persistent cough, shortness of breath, wheezing, sinus problems, or throat irritation since 9/11; 2) probable PTSD, which was assessed as in prior published Registry analyses with a cut-off score of 44 or greater on the PTSD Checklist, a September 11 specific PTSD checklist [31,32]; and 3) a self-reported post-9/11 physician diagnosis of at least one of the following chronic conditions: angina, asthma, hypertension, coronary heart disease, heart attack or other heart problems, stroke, emphysema, diabetes, and cancer, nearly all of which have been reported to be elevated among 9/11 exposed individuals [23,33-36]. The experience of any of this group of illnesses was chosen as the indicator because of the increasing public health importance of chronic diseases.

For assessment of bias in the prevalence of health outcomes measured in wave 2 or wave 3, and bias in the association between exposure and health outcomes, we selected the following health indicators: 1) self-assessed general health, classified as having poor or fair health versus excellent, very good, or good health; 2) recurrent lower respiratory symptoms (LRS), defined as in previous Registry publications as reporting shortness of breath, persistent cough, or wheezing for the first time at wave 1 and for 8 or more days in the last 30 days at the follow-up survey [27,28]; and 3) probable PTSD at wave 2 and wave 3, assessed by the same event-specific PTSD Checklist included in wave 1.

### Data analysis

We compared demographic characteristics, study recruitment source, eligibility group, disaster exposure, and wave 1 health status of wave 3 participants (wave 3 drop-ins and three-wave participants) with non-participants (wave 3 drop-outs and those who participated in wave 1 only). The adjusted odds ratios (AOR) and 95% confidence intervals (CI) for the association between wave 3 participation and each of these variables were computed using logistic regression.

We studied associations between wave 3 drop-out status (wave 3 drop-outs versus three-wave participants) and wave 2 health outcomes, using logistic regression models that controlled for demographics, recruitment source, eligibility group, disaster exposure, and survey mode. We used the same methods to study the associations between wave 3 drop-in status (wave 3 drop-ins versus three-wave participants) and wave 3 health outcomes.

We focused on the associations of exposure with two health outcomes, namely probable PTSD and LRS. These outcomes were measured at wave 2 for wave 3 drop-outs or at wave 3 for wave 3 drop-ins. We analyzed the association between these outcomes and exposure using logistic regression models estimated separately for three-wave participants, wave 3 drop-ins, and wave 3 drop-outs. We also estimated pooled logistic regression models that included interaction terms of each exposure variable and either wave 3 drop-in or wave 3 drop-out status as compared to being a three-wave participant to test whether the relationship between disaster exposure and health outcomes differed by survey participation (not shown). In all of these models, we also adjusted for demographics, study recruitment source, eligibility group, and survey mode.

Analyses were conducted in SAS version 9.2 (SAS Institute Inc., Cary, North Carolina).

## Results

### Baseline characteristics and wave 3 survey participation

Of the 67,670 enrollees who were eligible for wave 3, 42,934 (63%) participated in that survey. Compared to wave 3 participants, non-participants were younger, more likely to be male, non-White, have a household income below $50,000 in 2002, and less than post-graduate education (Table 1). List-identified enrollees were more likely to not participate in wave 3 (e.g. 36.7% of non-participants and 26.3% of participants were list-identified, AOR = 1.7, 95% CI: 1.7-1.8). Residents, area workers or passersby on September 11 were more likely to not participate in wave 3 as compared to rescue and recovery workers.

**Table 1:** **Study population characteristics and their associations with dropping-out of wave 3**

	**Wave 3 participants**	**Wave 3 non-participants**	**Likelihood of dropping out of wave 3**	
	**(N = 42,934) %**	**(N = 24,736) %**	**AOR** ^a^	**95% CI**
**Sex**				
Male	61.1	58.8	1.1***	1.1, 1.2
Female	38.9	41.2	reference	reference
**Age at wave 1 interview, year**				
<=24	3.5	6.7	2.2***	1.9, 2.5
25 - 44	46.2	56.0	1.8***	1.7, 2.0
45 - 64	45.9	32.7	1.0	0.9, 1.1
> = 65	4.3	4.7	reference	reference
**Race**				
Non-Hispanic black	10.2	15.0	1.8***	1.7, 1.9
Hispanic	11.6	16.2	1.5***	1.4, 1.6
Asian	5.8	9.4	16***	1.4, 1.7
Other-multi-racial	3.5	5.4	1.3***	1.2, 1.5
Non-Hispanic white	68.8	54.0	reference	reference
**Total household Income in 2002, $**				
<25,000	9.1	15.2	1.3***	1.3, 1.4
25,000- < 50,000	19.7	25.1	1.1**	1.1, 1.2
50,000- < 75,000	21.9	19.8	0.9	0.9, 1.0
75,000- < 150,000	37.1	29.0	0.9**	0.9, 1.0
> = 150,000	12.3	10.9	reference	reference
**Education**				
High school and below	22.6	29.2	1.4***	1.3, 1.5
College	57.0	54.6	1.2***	1.1, 1.2
Post-graduate	20.4	16.2	reference	reference
**Study recruitment source**				
List-identified	26.3	36.7	1.7***	1.7, 1.8
Self-identified	73.7	63.4	reference	reference
**Eligibility group**				
Lower Manhattan resident	14.0	20.0	1.6***	1.5, 1.7
Lower Manhattan area worker	34.0	33.1	1.2***	1.1, 1.2
Passersby on 9/11	5.1	6.1	1.3***	1.2, 1.4
Other^b^	0.2	0.2	0.8	0.5, 1.2
Rescue/recovery worker	46.6	40.7	reference	reference

Abbreviations: AOR, adjusted odds ratio; CI, confidence interval.

*P < 0.05; **P < 0.01; ***P < 0.0001.

^a^Adjusted for all factors listed in this table and in Table 2.

^b^Students and school staff in public schools south of Canal Street on 9/11/2001.

Regarding health status (Table 2), enrollees who did not participate in wave 3 were slightly more likely to have probable PTSD at wave 1 (17.9% as compared to 15.2% of non-participants, AOR =1.1, 95% CI: 1.1-1.2) and slightly less likely to have new or worsening respiratory symptoms since 9/11 (65.9% vs. 68.0%). Post-disaster chronic conditions were not related to wave 3 survey participation. None of the three selected measures of disaster exposure were associated with wave 3 non-participation.

**Table 2:** **Wave 1 health and disaster exposure and their associations with dropping-out of wave 3**

	**Wave 3 participants**	**Wave 3 non-participants**	**Likelihood of dropping out of wave 3**	
	**(N = 42,934) %**	**(N = 24,736) %**	**AOR** ^a^	**95% CI**
**Health at wave 1 (Baseline)**				
New or worsening respiratory symptoms since 9/11^b^				
Yes	68.0	65.9	1.0*	0.9, 1.0
No	32.1	34.2	reference	reference
PTSD > =44				
Yes	15.2	17.9	1.1***	1.1, 1.2
No	84.8	82.1	reference	reference
Chronic diseases diagnosed since 9/11^c^				
Yes	10.9	10.2	1.0	0.9, 1.0
No	89.2	89.8	reference	reference
**Disaster exposure**				
Witnessed traumatic or horrific event on 9/11/01				
Yes	37.1	37.6	1.0	1.0, 1.1
No	62.9	62.4	reference	reference
Sustained injury on 9/11/01				
Yes	13.7	12.5	1.0	0.9, 1.0
No	86.3	87.5	reference	reference
Caught in dust cloud on 9/11/01				
Yes	52.1	51.1	1.0	0.9, 1.0
No	47.9	49.0	reference	reference

Abbreviations: AOR, adjusted odds ratio; CI, confidence interval.

*P < 0.05; **P < 0.01; ***P < 0.0001.

^a^Adjusted for all factors listed in this table and in Table 1.

^b^Enrollees had developed one or more new or worsening respiratory symptoms (persistent cough, shortness of breath, wheezing, sinus problems, and throat irritation) since 9/11.

^c^Enrollees reported physician diagnosis of one or more of the diseases (angina, asthma, hypertension, coronary heart disease, heart attack or other heart problems, stroke, emphysema, diabetes, and cancer) since 9/11.

### Health outcomes of drop-ins/outs and three-wave participants at follow-up surveys

Wave 3 drop-outs and wave 3 drop-ins reported poorer health outcomes than the three-wave participants (Table 3). For example, 24.7% of the wave 3 drop-outs rated their health as poor or fair at wave 2 as compared to 19.4% of the three-wave participants. After adjusting for risk factors, the odds of reporting poor or fair health at wave 2 was 1.4 (95% CI: 1.3-1.4) times higher for wave 3 drop-outs than three-wave participants (Table 4). Compared to three-wave participants, wave 3 drop-outs had an odds ratio of 1.2 for probable PTSD (95% CI: 1.2-1.3) and 1.2 for recurrent LRS (95% CI: 1.1-1.3) at wave 2.

**Table 3:** **Comparison of health conditions at follow-up surveys among drop-ins/outs and three-wave participants**

		**Poor/fair health at wave 2 (%)**	**PTSD > =44 at wave 2 (%)**	**Recurrent LRS** ^a^ **at wave 2 (%)**
**Wave 2 participants (n = 46,120)**	Wave 3 drop-outs (n = 9,868)	24.7	21.6	22.4
	Three-wave participants (n = 36,252)	19.4	18.9	20.6
		**Poor/fair health at wave 3 (%)**	**PTSD > =44 at wave 3 (%)**	**Recurrent LRS at wave 3 (%)**
**Wave 3 participants (n = 42,934)**	Wave 3 drop-ins (n = 6,682)	33.7	22.4	18.6
	Three-wave participants (n = 36,252)	25.9	16.5	16.4

Abbreviations: PTSD, probable posttraumatic stress disorder; LRS, lower respiratory symptoms.

^a^Enrollees who reported at least 1 of 3 symptoms - shortness of breath, persistent cough, or wheezing - for the first time at wave 1 (i.e., post-9/11) and for 8 or more days (consecutive or nonconsecutive) in the last 30 days at wave 2 or wave 3.

**Table 4:** **Association between survey participation and enrollees’ health conditions**

	**Poor/fair health at wave 2**		**PTSD > =44 at wave 2**		**Recurrent LRS** ^**b**^ **at wave 2**	
Wave 3 drop-outs (N = 9,868)	AOR^a^	95% CI	AOR^a^	95% CI	AOR^a^	95% CI
	1.4***	1.3, 1.4	1.2***	1.2, 1.3	1.2***	1.1, 1.3
Three-wave participants (n = 36,252)	reference					
	**Poor/fair health at wave 3**		**PTSD > =44 at wave 3**		**Recurrent LRS** ^**b**^ **at wave 3**	
Wave 3 drop-ins (N = 6,682)	AOR^a^	95% CI	AOR^a^	95% CI	AOR^a^	95% CI
	1.3***	1.2, 1.4	1.4***	1.3, 1.5	1.2***	1.1, 1.3
Three-wave participants (n = 36,252)	reference					

Abbreviations: AOR, adjusted odds ratio; CI, confidence interval; PTSD, probable posttraumatic stress disorder; LRS, lower respiratory symptoms.

*P < 0.05; **P < 0.01; ***P < 0.0001.

^a^Adjusted for study recruitment source, sex, age, race, income, eligibility group, education, survey mode, and wave 1 exposure.

^b^Enrollees who reported at least 1 of 3 symptoms - shortness of breath, persistent cough, or wheezing - for the first time at wave 1 (i.e., post-9/11) and for 8 or more days (consecutive or nonconsecutive) in the last 30 days at wave 2 or wave 3.

Similarly, wave 3 drop-ins had higher odds of resporting less favorable health outcomes than three-wave participants at wave 3. The odds ratios for poor or fair health, probable PTSD, and recurrent LRS among wave 3 drop-ins as compared to three-wave participants were 1.3 (95% CI: 1.2-1.4), 1.4 (95% CI: 1.3-1.5), and 1.2 (95% CI: 1.1-1.3) respectively (Table 4). However, there were no health differentials between three-wave participants and W3 drop-ins/outs for other important health measures, including chronic bronchitis, gastroesophageal reflux disease (GERD), high cholesterol, asthma, hypertension, and anxiety (results not shown).

### Association between disaster exposure and health outcomes at follow-up surveys

**Figure 2:**
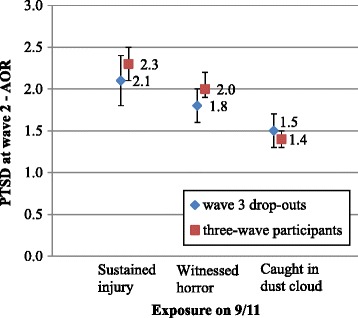
**Association between exposure and wave 2 PTSD - comparing wave 3 drop-outs with three-wave participants.**

**Figure 3:**
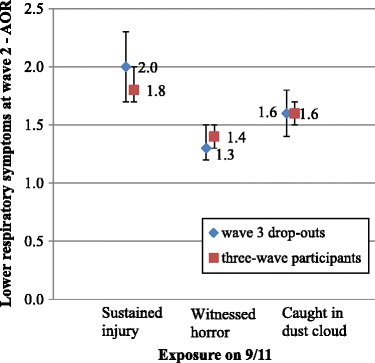
**Association between exposure and wave 2 LRS - comparing wave 3 drop-outs with three-wave participants.**

The association between disaster exposure and health outcomes did not differ substantially, either in significance or magnitude, among wave 3 drop-outs/drop-ins as compared to three-wave participants. For example, the magnitude of the association of exposure and wave 2 PTSD for wave 3 drop-outs and three-wave participants were almost identical (AORs 2.1 vs. 2.3, 1.8 vs. 2.0 and 1.5 vs. 1.4 for injury, witnessing horror and being caught in the dust cloud on 9/11, respectively) with overlapping 95% confidence intervals (Figure 2). The association of exposure measures and wave 2 LRS was also comparable between wave 3 drop-outs and three-wave participants, with similar adjusted odds ratios and overlapping 95% confidence intervals (Figure 3).

**Figure 4:**
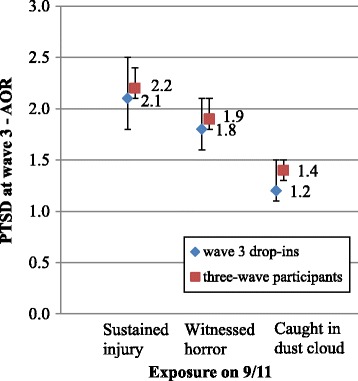
**Association between exposure and wave 3 PTSD - comparing wave 3 drop-ins with three-wave participants.**

**Figure 5:**
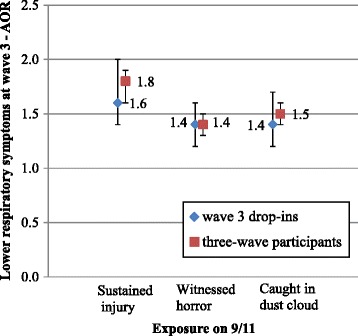
**Association between exposure and wave 3 LRS - comparing wave 3 drop-ins with three-wave participants.**

The comparison between wave 3 drop-ins and three-wave participants using the same health measures at wave 3 demonstrates the same patterns (Figures 4 and 5). The magnitude of the associations between disaster exposures and wave 3 PTSD and LRS were similar between wave 3 drop-ins and three-wave participants, with almost identical adjusted odds ratios and overlapping 95% confidence intervals. Using the estimated regression coefficients and their estimated standard errors, statistical significance tests were performed and the test results confirmed that the associations between disaster exposure and health outcomes as illustrated in Figures 2, 3, 4 and 5, among wave 3 drop-outs/drop-ins as compared to three-wave participants, were not statistically different (not shown).

In pooled models, there were no statistically significant interactions between 9/11 exposure and survey participation in their effects on either wave 2 or wave 3 health outcomes (not shown).

## Discussion

Attrition from the WTC Health Registry was not a substantial source of bias in the association between 9/11 disaster exposure and key physical and mental health outcomes such as probable PTSD and LRS. Specifically, the association between disaster exposure and probable PTSD and recurrent LRS was similar in magnitude and did not differ statistically between individuals who completed all three Registry surveys and those who either participated in wave 2 but not wave 3 (wave 3 drop-outs) or those who participated in wave 3 but not wave 2 (wave 3 drop-ins). The magnitude of the associations of disaster exposure and probable PTSD or recurrent LRS for wave 3 drop-ins and wave 3 drop-outs were within the range of earlier findings. For comparison, previously published estimates based on *all* wave 2 participants showed that the adjusted odds ratios for the association of probable PTSD with 9/11 injury ranged from 1.9 to 2.3 for different eligibility groups [23]. The similarity of the exposure and health association between drop-ins and drop-outs as compared to three-wave participants was evident despite the fact that three-wave participants reported better health in follow-up surveys.

This study also examined the associations between wave 1 characteristics and the likelihood of dropping out from the most recent follow-up survey (wave 3). Consistent with findings of earlier studies we found that non-participants of wave 3 were more likely to be younger, male, and of lower socioeconomic status [3-9]. Disaster exposures on September 11, 2001, including sustaining an injury, witnessing horror or trauma, and being caught in the dust cloud, were not significantly associated with loss to follow-up in the wave 3 survey. These findings are consistent with previous studies in which non-response was not strongly associated with disaster exposure or experience [13,15,37,38].

The association between non-response to a follow-up survey and two important baseline health indicators (probable PTSD and respiratory symptoms) was modest, and neither consistently positive nor negative. Although enrollees with probable PTSD at wave 1 were more likely to not participate in the follow-up, those with new and worsening respiratory symptoms since 9/11 were slightly less likely to drop out. A new post September 11 diagnosis of a range of chronic health conditions was not associated with attrition from wave 3. This is important because chronic health conditions will be a growing focus of Registry research as the population ages.

Although there were no consistent differences in initial wave 1 health status between wave 3 participants and non-participants, the wave 3 respondents who participated in all three Registry surveys were less likely to report poor or fair health, probable PTSD, or recurrent LRS at both wave 2 and wave 3 than those who participated in either wave 2 or wave 3 but not both. Therefore, omission of wave 2 and wave 3 non-participants in future analyses may lead to a downward bias in the prevalence of self-assessed poor or fair health, probable PTSD, or recurrent LRS. However, as indicated earlier, health differentials between three-wave participants and wave 3 drop-ins/outs were not present for other important health measures, including chronic bronchitis, GERD, high cholesterol, asthma, hypertension, and anxiety. Therefore, the downward bias in prevalence estimates arising from omission of wave 2 and wave 3 non-participants in future analyses will depend on the indicator being analyzed but should be minimal for a wide range of health indicators. In addition, as our results have shown, the small bias in prevalence estimates is not substantial enough to affect the positive association between 9/11-related exposure and health outcomes of major importance.

This study takes advantage of health information provided by survey drop-ins and drop-outs at the two follow-up surveys but does not provide health estimates directly for the non-participants who never responded to either of the two follow-up studies (wave 1 only participants). The lack of health data at the follow-up surveys for this group may affect our conclusions on non-response bias in prevalence estimates and in exposure-outcome association estimates, especially if these wave 1 only participants are remarkably different from the wave 3 drop-ins and drop-outs (one-time non-participants). However, this is unlikely the case for the WTC Health Registry cohort. Our analysis demonstrates that wave 3 drop-outs and drop-ins were more similar to wave 1 only participants than to three-wave participants on key demographic measures (sex, age, race, income, and education) and on study recruitment source (results not shown).

The WTC Health Registry is an epidemiological cohort study of distinct groups of disaster survivors that share little in common besides exposure to the 9/11 terrorist attack. To follow up and to understand the characteristics of such a large, diverse, and dynamic disaster cohort requires substantial efforts. We suggest that efforts to maximize participation in future follow-up surveys should focus on lower Manhattan residents, area workers, passersby, and list-identified enrollees. These efforts along with a number of other ongoing strategies such as tracing enrollees lost to follow-up, sharing Registry findings and recommendations with enrollees by distributing an annual report, and personalized outreach to refer enrollees treatment resources will help the WTC Health Registry continue successfully as a long-term prospective cohort.

The WTC Health Registry is an essential component of the public health response to a major disaster. Since 2002, the Registry has served as a platform for numerous 9/11-related disaster studies that have helped define the extent of the physical and mental health impact of the disaster for thousands of people [23,27,33,34,36,39]. These extensive epidemiological findings, coupled with an active outreach program within the Registry, have helped to engage enrollees and their families with the extensive network of 9/11 health care providers that was created as part of the disaster response [40]. This non-response bias evaluation study reveals that, despite all efforts demonstrated to boost response rate, prevalence estimates may be underestimated in the follow-up studies. Nonetheless, the associations of disaster exposures and health outcomes are highly consistent with findings from many other studies of physical and mental health outcomes in 9/11 exposed individuals [41].

## Conclusion

Non-response bias has been a concern since the inception of the WTC Health Registry [42]. As time goes on and chronic diseases emerge, it becomes increasingly important to conduct a careful assessment of non-response bias in the follow-up studies. Few longitudinal studies of disaster cohorts have examined both selective attrition and bias in prevalence estimates of health outcomes. Our study addresses both issues and further assesses the extent to which non-response may have affected estimates of associations between health outcomes and disaster exposures. Our results show that, despite a somewhat downward bias in prevalence estimates, attrition from the WTC Health Registry follow-up studies does not lead to serious bias in associations between 9/11 disaster exposures and key health outcomes. In doing so, the results of this study provide a level of confidence in overall WTC Health Registry findings.

## Abbreviations

WTC: World Trade Center
PTSD: Posttraumatic stress disorder
CATI: Computer Assisted Telephone Interviewing
LRS: Lower respiratory symptoms
AOR: Adjusted odds ratio
CI: Confidence interval
GERD: Gastroesophageal reflux disease
